# High-flexibility combinatorial peptide synthesis with laser-based transfer of monomers in solid matrix material

**DOI:** 10.1038/ncomms11844

**Published:** 2016-06-14

**Authors:** Felix F. Loeffler, Tobias C. Foertsch, Roman Popov, Daniela S. Mattes, Martin Schlageter, Martyna Sedlmayr, Barbara Ridder, Florian-Xuan Dang, Clemens von Bojničić-Kninski, Laura K. Weber, Andrea Fischer, Juliane Greifenstein, Valentina Bykovskaya, Ivan Buliev, F. Ralf Bischoff, Lothar Hahn, Michael A. R. Meier, Stefan Bräse, Annie K. Powell, Teodor Silviu Balaban, Frank Breitling, Alexander Nesterov-Mueller

**Affiliations:** 1 Karlsruhe Institute of Technology, Institute of Microstructure Technology (IMT), Hermann-von-Helmholtz-Platz 1, 76344 Eggenstein-Leopoldshafen, Germany; 2 Karlsruhe Institute of Technology, Institute of Organic Chemistry (IOC), Fritz-Haber-Weg 6, 76131 Karlsruhe, Germany; 3 Karlsruhe Institute of Technology, Institute for Inorganic Chemistry, Engesserstrasse 15, 76131 Karlsruhe, Germany; 4 Karlsruhe Institute of Technology, Institute of Nanotechnology (INT), Hermann-von-Helmholtz-Platz 1, 76344 Eggenstein-Leopoldshafen, Germany; 5 Aix-Marseille University, CNRS UMR 7313, Institut des Sciences Moléculaires de Marseille, Chirosciences, 13397 Marseille cedex 20, France; 6 Technical University of Varna, 1 Studentska, 9010 Varna, Bulgaria; 7 German Cancer Research Center, INF 580, 69120 Heidelberg, Germany; 8 Karlsruhe Institute of Technology, Institute of Toxicology and Genetics (ITG), Hermann-von-Helmholtz-Platz 1, 76344 Eggenstein-Leopoldshafen, Germany

## Abstract

Laser writing is used to structure surfaces in many different ways in materials and life sciences. However, combinatorial patterning applications are still limited. Here we present a method for cost-efficient combinatorial synthesis of very-high-density peptide arrays with natural and synthetic monomers. A laser automatically transfers nanometre-thin solid material spots from different donor slides to an acceptor. Each donor bears a thin polymer film, embedding one type of monomer. Coupling occurs in a separate heating step, where the matrix becomes viscous and building blocks diffuse and couple to the acceptor surface. Furthermore, we can consecutively deposit two material layers of activation reagents and amino acids. Subsequent heat-induced mixing facilitates an *in situ* activation and coupling of the monomers. This allows us to incorporate building blocks with click chemistry compatibility or a large variety of commercially available non-activated, for example, posttranslationally modified building blocks into the array's peptides with >17,000 spots per cm^2^.

## Abstract (web summary)

Peptide arrays are used in areas such as measuring protein-protein interactions, but achieving high density in synthesis is challenging. Here, the authors report a method for the combinatorial synthesis of high density peptides arrays by laser driven sequential transfer of monomers onto acceptor surfaces.

## Graphical abstract

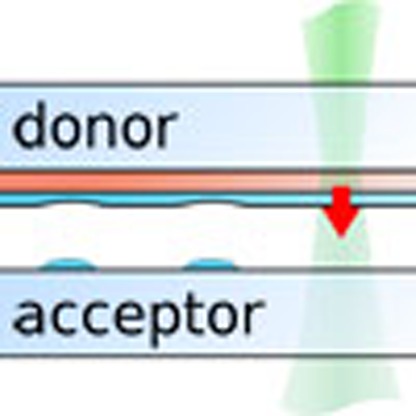

Laser direct-write approaches allow for versatile two- and three-dimensional structuring of a given workpiece in many different ways^1^,^2^,^3^. Laser-induced forward transfer (LIFT), a variant of the direct-write processes, uses laser irradiation to transfer liquid or solid material from a donor surface to defined areas of an acceptor surface. Thereby, a workpiece can be microstructured with different material patterns by simply employing several donor surfaces consecutively. The LIFT method and its variants are applied in the production of, for example, Samsung's organic light-emitting diode display^4^ and other electronic materials and devices^5^,^6^,^7^, nanoparticle^8^ or hydrophobic/hydrophilic surface pattern generation^9^, as well as several biological patterning applications^10^, such as hydrogels^11^, biomolecules^12^,^13^,^14^ and even cells^15^,^16^. Very small feature sizes of down to 3 μm were reported^17^. This versatility sparked the idea to combine LIFT with combinatorial chemistry, where a number of building blocks are used to synthesize a vast number of different oligomeric molecules.

Most of today's array-based combinatorial peptide chemistry methods rely on Merrifield's solid-phase synthesis^18^: the SPOT synthesis^19^, the light-directed lithographic synthesis^20^,^21^,^22^,^23^,^24^, the electro-chemical synthesis^25^ and the particle-based synthesis^26^,^27^,^28^,^29^. In the SPOT method, peptides are elongated in parallel on an array by spotting 20 different dissolved and chemically activated amino-acid building blocks at discrete locations. The light-directed lithographic synthesis uses light patterns to remove photolabile protecting groups from the growing peptides or photogenerated acids cleaving the acid-labile protecting groups at selected areas with a digital micromirror device. Yet, these methods have severe drawbacks: liquid solvents in the SPOT synthesis tend to evaporate or spread on the surface, which limits the spot density of arrays to some 25 peptides per cm^2^. Lithographic methods offer a much higher density, but they allow for only one type of amino-acid building block to be coupled sequentially to the array, which yields lower synthesis quality and necessitates, for example, 200 coupling reactions to synthesize arrays of 10mer peptides using 20 different amino acids (10 × 20).

The particle-based variant of the Merrifield synthesis method uses, for example, a laser printer or a microelectronic chip to structure a surface with 20 different types of solid polymer particles, each embedding a different type of amino-acid building block. This approach overcomes most of the mentioned drawbacks: structuring is achieved by electrical fields to precisely deposit patterns of the 20 different amino acid particle types, before the coupling reaction is induced for the whole pattern at once by heating. At 90 °C, the solid matrix material becomes viscous and the spots are transformed into gel-like droplets that serve as spatially confined reaction vessels, without evaporation or spreading on the surface. This enables the defined diffusion and coupling of the pre-activated monomers to free amino groups on the surface. However, some inherent drawbacks still remain: (i) the spot density is still not satisfying; (ii) the number of different amino-acid building blocks is limited due to process restrictions and high material consumption; and (iii) due to the rather large particle size and the slow diffusion within the melted matrix material, it is almost impossible to employ the large variety of commercially available non-activated amino-acid building blocks by *in situ* activation.

Our goal was to overcome the aforementioned problems with a combinatorial LIFT method (cLIFT), which allows us to synthesize affordable, very high-density (>17,000 spots per cm^2^) and high-quality peptide arrays. Furthermore, its high flexibility enables us to employ a large variety of commercially available non-activated amino-acid building blocks and, thereby, synthesize peptide arrays with, for example, many different posttranslationally modified peptides. We developed an automated machine setup, including a two-dimensional laser scanning system, which can currently accommodate over a hundred different donor surfaces. The laser rapidly and accurately transfers very small-sized and very thin material spots next to each other or on top of each other to defined locations on an acceptor surface. Each material spot can comprise a different amino-acid building block or the chemicals that are needed for an *in situ* activation of a non-activated amino-acid building block.

## Results

### Principle

**Figure 1:**
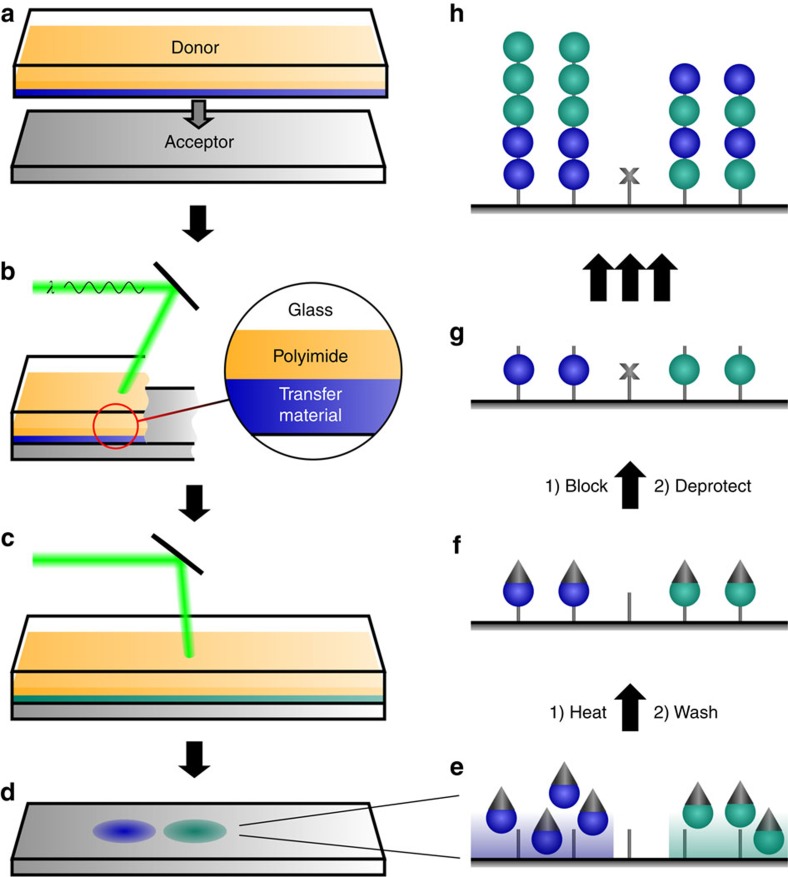
Principle of the combinatorial laser-induced forward transfer synthesis. Donor slides bearing different monomer building blocks, embedded in a resin (different colours in **b** and **c**) are positioned on top of an acceptor slide (**a**). A laser scanning system transfers minute amounts of material to the acceptor slide. Repeating these steps with different donor slides results in a pattern of different amino acid types (**d**). The coupling reaction of monomers (**e**) is initiated by heating the surface. Next, uncoupled amino-acid building blocks are removed (**f**), uncoupled amino groups are blocked and then the protecting groups are removed (**g**). Repeating the cycle generates an array of combinatorially synthesized peptides (**h**).

The principle of the cLIFT method is shown in Fig. 1. The method requires an acceptor slide and different donor slides, which are the sources of the different amino-acid building blocks (Fig. 1a). Donor slides are composed of a standard microscope glass slide, which is covered by a light-absorbing self-adhesive polyimide foil with a thickness of ∼95 μm and a transfer material layer. The latter is generated by first dissolving a commercially available styrene-acrylic copolymer resin matrix and OPfp-activated amino acid or biotin building blocks in dichloromethane (DCM), followed by spin-coating this mixture on top of the polyimide foil. The solid matrix efficiently shields chemically activated amino-acid building blocks from decay^27^. The low-cost and easy-to-handle self-adhesive polyimide is attached to the glass slide's surface with a laminating machine.

As acceptor slides, we use commercially available amino-terminated poly (ethylene glycol) methacrylate (PEGMA)/methyl methacrylate 10/90 slides. For more details, please refer to the Methods section.

**Figure 2:**
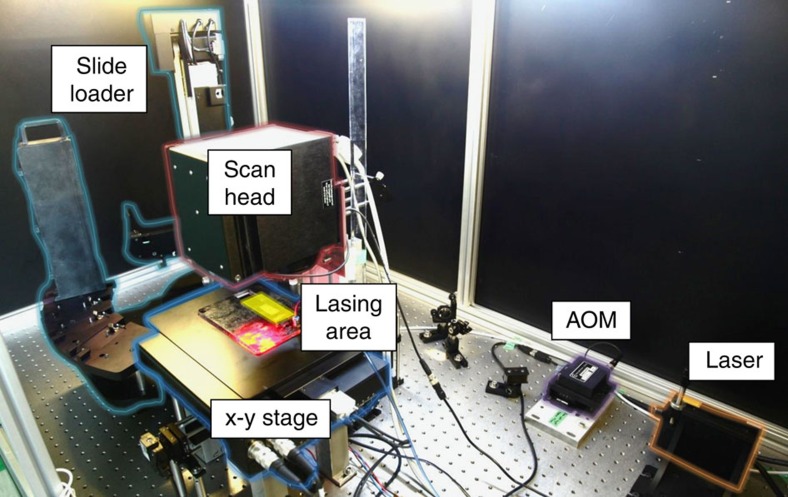
Setup of the combinatorial laser-induced forward transfer machine. The laser is modulated by an acousto-optic modulator (AOM) and guided to a scan head system. The laser transfer is conducted on an *x*–*y* microscope stage, the lasing area is highlighted in yellow. Donor and acceptor slides are automatically handled and placed by the robotic slide loader.

We automated the entire patterning procedure (see Fig. 2 and Supplementary Movie 1), schematically described in Fig. 1a–d. A robotic slide loader automatically handles the donor and acceptor slides, and positions the transfer layer of the donor slide directly on top of the N-terminated acceptor substrate as described in Fig. 1b. Next, selected spots of matrix material with a first type of embedded amino acid monomers are transferred to the acceptor slide by short laser pulses (Fig. 1c,d). Pulses are generated by an acousto-optic modulator, which rapidly switches the laser radiation of a 1-W continuous-wave 532 nm laser, whereas a commercially available laser scanning system directs the laser focus position (for details, see ‘cLIFT machine setup' in Methods). We investigated the material transfer in a wide range of laser pulse durations from micro to milliseconds (standard irradiation time per spot: 2–7 ms).

During the short laser transfer process, time and temperature do not suffice to initiate the coupling reaction of activated monomers to the substrate (data not shown). This is achieved by heating the patterned acceptor slide in an oven for 60 min to 90 °C (Fig. 1e). The heating step initiates the diffusion of the monomers within the matrix material, which allows them to couple to free amino groups on the acceptor slide (for details on the solid phase chemistry, see Supplementary Fig. 1). Subsequent processing steps are identical to standard solid-phase peptide synthesis procedures: first, we remove excess monomers and resin (Fig. 1f), block unreacted amino groups on the surface (‘capping'), remove the N-terminal 9-fluorenylmethoxycarbonyl (Fmoc) protecting groups and finally dry the acceptor slides (Fig. 1g). When repeated several times, we obtain an array of combinatorially synthesized peptides (Fig. 1h). We have also automated the chemical washing steps in a wet chemistry machine setup.

### Laser transfer

**Figure 3:**
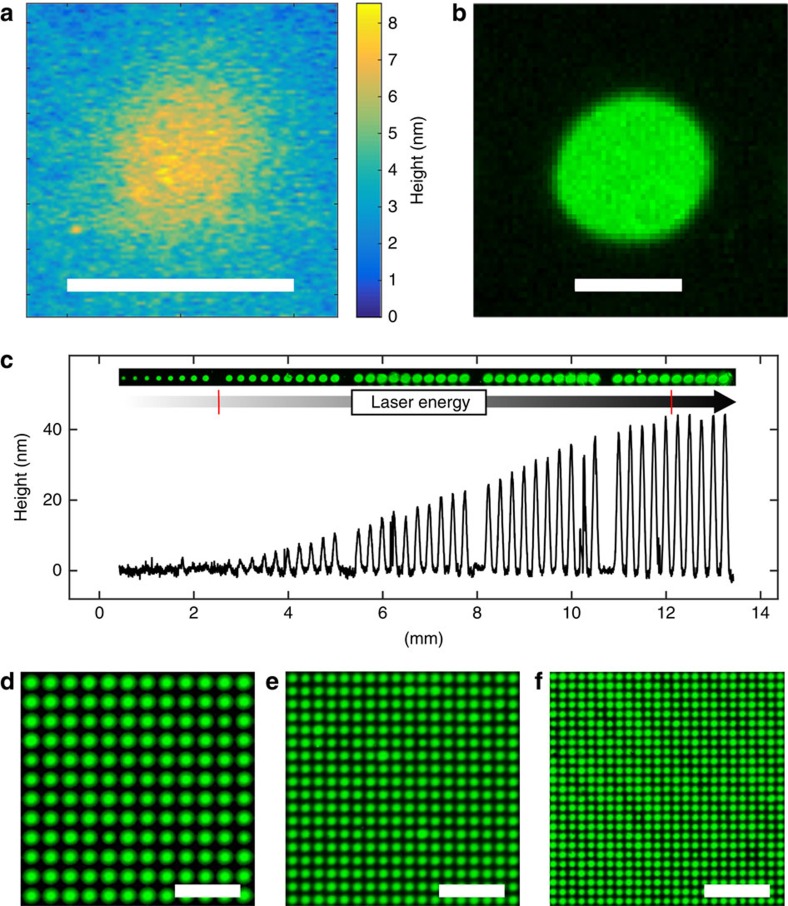
Analysis of transferred spots and spot resolution. (**a**) Topography of the spot with activated leucine monomers in the polymer matrix on the acceptor slide before the coupling and washing steps, measured with phase-shift interferometry. The material spot is ∼8 nm thick at its centre (scale bar, 100 μm). (**b**) The fluorescence image of the spot after coupling of the monomers and staining with a rhodamine dye. The amount of the transferred material is ∼0.1 ng (scale bar, 100 μm). (**c**) Topography of transferred spot material in dependence of the laser energy. The height of the transferred spot material, containing an activated leucine building block, was measured with phase-shift interferometry, laser energy linearly increases from left to right in steps of ∼15 μJ. The range of the linear correlation of deposited material (1–50 nm) and deposited laser energy (450–900 μJ) is marked by red lines. The corresponding fluorescence staining pattern of the coupled leucine was obtained with a rhodamine dye. (**d**–**f**) Fluorescence images of biotin patterns (scale bar, 500 μm), stained with labelled streptavidin, achieving different pitches: (**d**) 150 μm (4,444 spots per cm^2^), (**e**) 100 μm (10,000 spots per cm^2^), (**f**) 75 μm (17,777 spots per cm^2^).

Remarkably, and similar to laser ablation, laser-induced material transfer occurs through air over a distance of up to 60 μm (see Supplementary Methods and Supplementary Figs 2 and 3). A laser pulse is absorbed in the polyimide foil, where the energy is converted into heat, which causes the polyimide to quickly expand. Nevertheless, strong plastic deformations and blisters within the polyimide material are only observed when using very high-energy laser pulses (see Supplementary Fig. 4). In parallel, the heat diffuses also into the less rigid transfer layer, melting the material layer and evaporating residual solvent in the transfer layer, which causes the ejection and transfer of material. When analysing the transfer layer in those regions that ‘donated' material with atomic force microscopy, tiny impulse craters, and molten and re-solidified material can be observed (see Supplementary Figs 4 and 5). We also analysed the topography of the transferred material on the acceptor slide with a phase-shift interferometer and we found that the height of the transferred material spot on the acceptor slide is in the order of several nanometres (Fig. 3a). Analysing the coupled and stained amino acid monomers on the acceptor surface with a fluorescence scanner (Fig. 3b), we found that the spot dimensions approximately correspond to the crater size on the donor slide. Obviously, the material on the acceptor slide stems from the crater region that is visible on the donor slide.

Furthermore, by tuning the laser transfer parameters, we are able to adjust the amount of deposited material (see Fig. 3c). Interestingly, the amount of deposited material on the acceptor surface, which is approximately in the range between 1 and 50 nm in terms of layer thickness (10–500 pg), correlates linearly with the laser energy, ranging from 450 to 900 μJ (red marks in Fig. 3c). Above 900 μJ, the amount of deposited material reaches a plateau, until the laser starts to burn the polyimide layer. Below 400 μJ, almost no material is transferred, although a weak fluorescence staining is visible down to about 300 μJ.

Another feature of our approach is the possibility to reuse the donor slides up to 20 times without loss in transfer quality, making the cLIFT process highly efficient (see Supplementary Methods and Supplementary Figs 6 and 7).

To assess the feasibility of various spot pitches, we transferred OPfp-activated biotin to a functionalized glass substrate with cLIFT using different lasing parameters. Owing to the heat diffusion within the 95-μm-thick self-adhesive polyimide layer, which limits the lowest possible pitch to ∼100 μm, we replaced the very thick polyimide layer by a 5-μm thin layer of spin-coated and cured polyimide (Durimide 7520, Fujifilm). In this way, we achieved pitches from 150 to 75 μm (Fig. 3d–f). In principle, LIFT technology allows for very high-density patterns with resolutions of up to a few micrometres^17^. Details on the synthesis and staining experiments shown in Fig. 3 are described in ‘cLIFT technique parameters' and ‘General procedure' in Methods.

We used these experimental findings to derive an analytical heat diffusion model to define the conditions where laser-induced material transfer takes place (see Supplementary Methods and Supplementary Fig. 8). In a simplified model, we describe the heat diffusion in the light-absorbing layer. Laser pulse-induced heating competes with heat diffusion and conditions are tipped towards transfer conditions either by higher laser powers or by longer pulse durations. These transfer conditions are described by the formula:


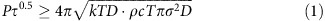


Here, *P* is the total laser power absorbed by the donor slide, *τ* is the pulse duration, *σ* is the laser focus radius, *D* is the thickness of the layers on the donor substrate, *T* is the characteristic temperature of the transfer material layer where the transfer can take place, *k* is the heat transfer coefficient, *ρ* is the mass density and *c* is the specific heat capacity.

### Synthesis

**Figure 4:**
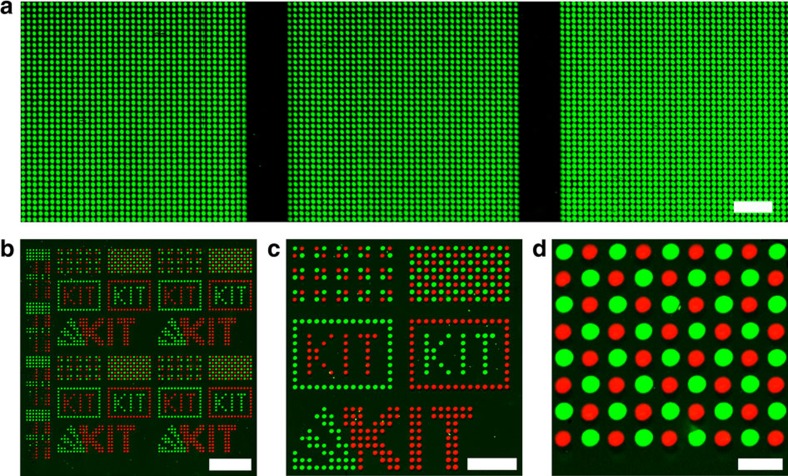
Combinatorial synthesis of peptide arrays with cLIFT. (**a**) Three spot patterns of a 3-, 6- and 9-mer peptide (left, centre and right) with a biotin momoner as a terminal group (left Ala-Gly-biotin, centre Ala-Gly-Ala-Gly-Ala-biotin and right Ala-Gly-Ala-Gly-Ala-Gly-Ala-Gly-biotin); scale bar, 1 mm. No significant difference in staining intensity can be observed. (**b**–**d**) Array containing 64 different peptides with 4,444 peptide spots per cm^2^ using cLIFT and different donor slides that bear the different amino-acid building blocks; scale bars, (**b**) 2 mm, (**c**) 1 mm, (**d**) 250 μm. Flag- and HA peptides (**c**, **d**) and 62 variants (**b**, left column) were stained with specific anti-Flag and anti-HA antibodies.

First, we used the cLIFT machine (Fig. 2) to synthesize patterns of 3-mer, 6-mer and 9-mer peptides, with an alternating sequence of Ala and Gly, and a terminal biotin (Fig. 4a). These arrayed peptides were stained with fluorescently labelled streptavidin. We did not observe any significant decrease in fluorescence intensity, when we compared the staining intensity of the 3-mer, 6-mer and 9-mer peptides (for statistical analysis of spot fluorescence signals, see Supplementary Methods and Supplementary Fig. 9).

Next, we synthesized haemagglutinin (HA) (Tyr-Pro-Tyr-Asp-Val-Pro-Asp-Tyr-Ala) and Flag epitopes (Tyr-Asp-Tyr-Lys-Asp-Asp-Asp-Asp-Lys), as well as 62 permutation variants of these peptides, by exchanging two amino acids in each of the six differing layers of the synthesized peptides (2^6^=64 variants). The selected peptides were synthesized in a pitch of 150 μm with measured spots sizes between 100 and 120 μm. Side-chain protecting groups were cleaved from the resulting peptides with a trifluoroacetic acid solution (for details, see ‘General procedure' and ‘Typical duration of synthesis for one layer' in Methods). Subsequently, the array was incubated with fluorescently labelled specific antibodies (Fig. 4b–d). Strong signals and negligible background indicate a good quality of the peptide spots, synthesized with cLIFT (for statistical analysis of spot fluorescence signals, see Supplementary Methods, Supplementary Fig. 10 and Supplementary Tables 1–4). Details on chemical analytics can be found in Supplementary Methods and Supplementary Figs 11–13. In our previous work, we did not observe any significant decay of activated monomers due to short laser irradiation^29^ with comparable laser energies.

**Figure 5:**
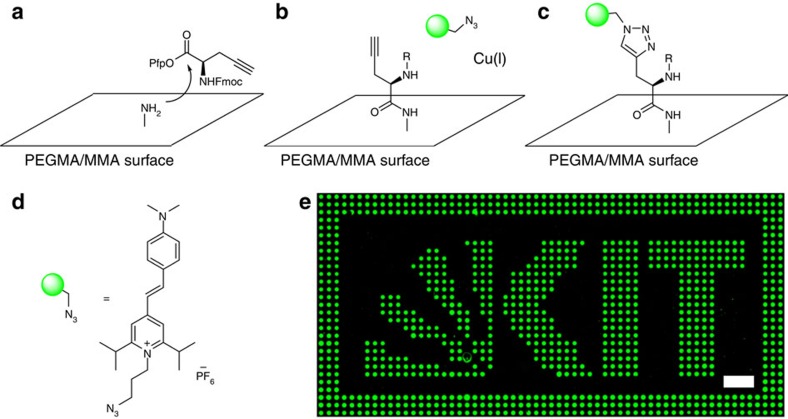
Schematic of the click chemistry reaction using an Fmoc protected propargyl-glycine-OPfp (Fmoc-Pra-OPfp). (**a**) The synthetic amino acid (Pra, propargyl-glycine) was patterned and coupled with cLIFT. (**b**–**d**) Subsequently, the styrylpyridinium fluorophore was coupled to the Pra in a copper catalysed click reaction. (**e**) The fluorescent image of the patterned Pra coupled with the styrylpyridinium fluorophore via click chemistry; spot pitch 250 μm (scale bar, 1 mm).

Click chemistry is of increasing interest in the synthesis of peptides and drug conjugates^30^,^31^. To demonstrate the chemical flexibility of cLIFT, we also patterned and coupled a synthetic amino acid on a large scale with an alkyne side group, a Fmoc-protected propargyl-glycine-OPfp to the amino-activated acceptor substrate (Fig. 5a). Then, we used a copper-catalysed click reaction to label this pattern with a styrylpyridinium fluorophore^32^, functionalized with an azide group (Fig. 5b–d). We observed a bright fluorescence pattern (Fig. 5e) and a very low background signal. Thus, we can presume that our laser transfer does not harm the alkyne function of the activated Pra monomer (details in Supplementary Methods). Furthermore, X-ray photoelectron spectroscopy (XPS) analysis of the surface showed no residual copper catalyst on the synthesis slide.

**Figure 6:**
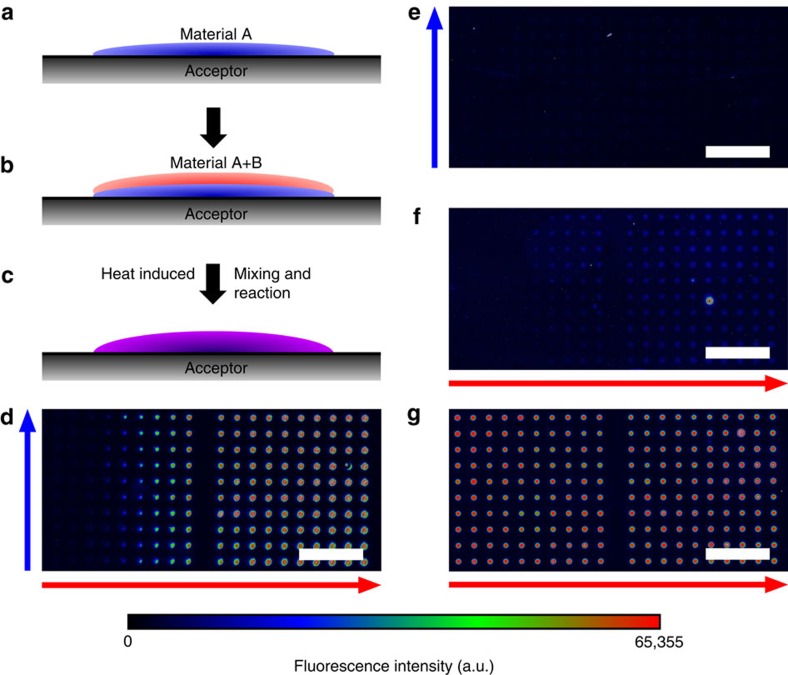
Two-step cLIFT gradient patterning experiment for solid-phase combinatorial synthesis. (**a**) Deposition of first material. (**b**) Deposition of a second material on top of the previous pattern. (**c**) Heat-induced melting of material induces mixing and initiates the reaction. (**d**–**g**) Experimental results of an Fmoc-Gly-OH, reacting with DIC and HOBt activation reagents (250 μm pitch); scale bar, 1 mm. Free amino groups were stained with a rhodamine *N*-hydroxysuccinimide (NHS) ester dye, illustrated in rainbow colour scale. (**d**) Two layer reaction of a layer of DIC and HOBt (concentration increases in direction of blue arrow) with a second layer of Fmoc-Gly-OH (concentration increases in direction of red arrow); (**e**) one layer of DIC and HOBt as a negative control; (**f**) one layer of Fmoc-Gly-OH as a negative control; (**g**) one layer of Fmoc-Gly-OPfp as a positive control.

Finally, we wanted to assess whether our cLIFT method can directly employ the plethora of commercially available Fmoc-protected, but non-activated, amino-acid building blocks. For the surface coupling reaction, building blocks have to be *in situ* activated. Therefore, we deposited material spots with non-activated amino acids on top of previously deposited material spots that contain suitable activation agents (Fig. 6a–c). First, we deposited material spots that contained a mixture of the activation reagent *N*,*N*'-diisopropylcarbodiimide (DIC) and the racemization suppressor hydroxybenzotriazole (HOBt), embedded in the solid matrix material (Fig. 6d,e). By increasing the lasing duration, we generated a gradient of increasing amounts of transferred activation reagents (blue arrow). Next, we positioned a second layer of material spots on top of the activation reagent spots, containing a non-activated glycine with an N-terminal protecting group (Fmoc-Gly-OH). Again, we generated a gradient of transferred materials by increasing the lasing time (gradient pattern from left to right, red arrow, perpendicular to the first layer; Fig. 6d). As controls, activation reagents only (Fig. 6e) and non-activated Fmoc-Gly-OH only (Fig. 6f) were used. An OPfp-ester-activated glycine (Fmoc-Gly-OPfp) was deposited as a positive control (Fig. 6g). After heat-induced coupling, we performed the washing and capping steps to block free amino groups on the surface and removed the Fmoc-protecting groups from the surface-bound amino acids. Then, we coupled a rhodamine *N*-hydroxysuccinimide ester dye to the free NH_2_ groups on the array. We observed a strong fluorescent signal in those spots where non-activated amino acids were positioned on top of the activation reagents (Fig. 6d), but no or very weak signals for the two negative controls with only DIC/HOBt (Fig. 6e) and only Fmoc-Gly-OH (Fig. 6f). These observations indicate that non-activated amino-acid building blocks are efficiently converted to active esters, when diffusing through a very thin layer of melted activation reagents. As expected, the positive control with activated Fmoc-Gly-OPfp (Fig. 6g) also gave strong fluorescent signals. We presume that the weak fluorescent signals, which are only observed in the negative control of Fmoc-Gly-OH, are either due to nonspecific intercalation of the Fmoc-Gly-OH building block into the PEGMA/methyl methacrylate surface or result from a rare peptide bond formation of the non-activated glycine with the amino groups on the surface, induced by the high coupling temperature (for experimental details, see ‘Preparation of the donor and acceptor slides' and ‘Activation reaction' in Methods). Thus, exploiting the cLIFT method, it is possible to separately pattern and precisely mix two or more types of reagents on the surface. By adjusting laser transfer (Fig. 3c) and spin-coating parameters (that is, initial concentration of reagents), it is also easily possible to predetermine the amounts and, thereby, the concentrations of the monomers and activation agents.

## Discussion

Life sciences strive to find out which molecules bind to each other, for example, which antigens are targeted by an antibody or which posttranslational modifications are crucial in specific cell signalling, for example, which acetyl-lysine-peptides are bound by a bromodomain^33^. One straightforward method to find out is the use of high-density peptide arrays, but all currently available methods to synthesize them have severe drawbacks: lithographic methods yield only short, low-quality peptides, due to the large number of coupling cycles. The SPOT synthesis yields high-quality, but low-density peptide arrays, due to solvents that tend to evaporate and spread on the surface. Particle-based methods yield medium-density peptide arrays that are limited to only 24 different building blocks, due to the limitation of the number of particle types and toner cartridges. However, simply by exchanging one donor foil for a different one, our cLIFT method can employ a theoretically unlimited number of different amino-acid building blocks. Moreover, the automated cLIFT machine does not require any expensive mechanical alignment, but rather relies on an inexpensive camera system, which reliably calibrates the laser beam with reference markers on the array surface (see Supplementary Figs 14 and 15). Thereby, we can currently synthesize arrays with a density of >17,000 spots per cm^2^, which is certainly not the limit in terms of achievable array densities. It has been reported that LIFT-based structuring is possible with feature sizes of down to 3 μm^17^. Yet, another major advantage of our cLIFT method is its frugal consumption of expensive amino-acid building blocks: a few milligrams suffice to produce a donor slide, whereas the amount of transferred material is in the nanogram range. In particular, donor slides with expensive building blocks can be reused at least up to 20 times.

Although the exact mechanism of our laser-induced material transfer is still elusive, we could show that the size and the thickness of transferred spots can be adjusted by tuning the laser parameters. We exploited this feature by positioning two different and very thin material spots on top of each other, which consistently mixed on melting. As cLIFT structuring is performed with solid materials, both the *in situ* activation and the peptide elongation reaction are inhibited (‘frozen'), until the heating step initiates the reaction by melting the material spots. This feature allows for structuring with many different materials that are positioned next to or on top of each other. After these structuring steps, heat-induced melting makes it possible for the amino acid building blocks to diffuse, the activation reagents to activate the amino acids and finally for the coupling of the activated amino acids to the growing peptides spot-by-spot on the array. As it is possible to control the amount of deposited materials, any combination of different reagents for peptide synthesis should be feasible. Thus, our method profits from an ever increasing number of commercially available Fmoc-protected amino-acid building blocks to synthesize, for example, peptides with different types of posttranslational modifications. Furthermore, we were able to include a synthetic amino-acid building block, showing that advanced postsynthesis functionalization (that is, click chemistry) is compatible with our synthesis approach. Currently, we have advanced our cLIFT method to nearly complete automation: we integrated a slide loader to automatically exchange donor slides, whereas wet-chemistry processing is done in a fully automated ultrasound-supported reactor.

## Methods

### Preparation of the donor and acceptor slides

Donor slide preparation: microscope glass substrates were covered by self-adhesive polyimide foil (Kapton, DuPont, USA; cmc Klebetechnik GmbH, Frankenthal/Pfalz, Germany; thickness of polyimide layer ∼50 μm, thickness of glue layer ∼45 μm). The transfer material layer, similar to other solid material-based synthesis methods^26^,^27^,^29^, was spin coated (80 r.p.s. for 45 s; solution was already applied during acceleration to 80 r.p.s.) on top of the polyimide foil. For this purpose, 10% w/w activated monomers, for example, pentafluorophenyl (OPfp)-activated amino acids with an N-terminal Fmoc-protecting group, and 90% w/w of the inert matrix polymer (SLEC PLT 7552, Sekisui Chemical GmbH, Düsseldorf/Germany) are dissolved in DCM: 15 mg of amino acid and 135 mg of resin are dissolved in 1 ml of DCM, or 8 mg of biotin and 68 mg of resin in 1 ml of DCM, respectively, due to the lower solubility of biotin in DCM. In the case of the coupling reagent donor slide, we dissolved 3.75 mg of HOBt in 12 μl of dry *N*,*N*-dimethylformamide (DMF) and then added 988 μl of DCM, 139 mg of matrix polymer and 9.35 μl (7,25 mg) of DIC. The donor slide with the non-activated glycine amino acid (Fmoc-Gly-OH) was prepared by dissolving 15 mg of Fmoc-Gly-OH in 25 μl of DMF and then adding 975 μl of DCM and 135 mg of matrix polymer.

Acceptor slides: The PEGMA slides were acquired from PEPperPRINT GmbH, Germany. The top surface of the acceptor slide was marked by laser ablation with a high-power laser^34^. The marks were used for determining the position of the acceptor slides with respect to the laser scanning system.

### cLIFT machine setup

A robotic slide loader (PL200, Prior Scientific, UK) automatically handles and places the donor and acceptor slides. We use an acousto-optic modulator (1002AF1, Polytec GmbH, Germany) to switch the laser (FSDL-532-1000T, 1 W, Frankfurt Laser Company), a laser scanning system (hurrySCAN 10, Scanlab AG, Germany), an *x*–*y* microscope stage (SCANplus 100 × 100, Maerzhaeuser, Germany) and a camera (DCC1645C, Thorlabs Inc., Newton, NJ, USA) with a microscope lens (PLN 4XCY, Olympus GmbH, Hamburg, Germany).

### cLIFT technique parameters

For a pitch of 150 μm, we used the full power of the 1 W laser and a pulse duration of 5 ms. The scan head was set to a jump speed of 100 μs, a laser-off delay of 310 μs, a jump delay of 200 μs and a laser-on delay of 300 μs.

### General procedure

After the patterning of one layer with different monomers, the coupling reaction is initiated by heating the acceptor slide in an oven to 90 °C for 60 min under argon atmosphere. Next, the acceptor slide was washed with acetone three times for 2 min (once in an ultrasonic bath). Then, the acceptor slide was dried in a jet of air. For the HA- and Flag-peptide synthesis, the patterning and coupling steps were repeated to increase the coupling yield.

To block the remaining free NH_2_ groups on the acceptor slide, it was washed in a mixture of acetic anhydrate (Ac_2_O, 10%), *N*,*N*-diisopropylethylamine (DIPEA, 20%) and DMF (70%) for 30 min. Then, the slide was washed twice for 5 min with DMF.

The deprotection of NH_2_ groups of the terminal amino acids was performed by washing the slide in a solution of piperidine (20%) and DMF (80%) for 20 min. Afterwards, the acceptor slide was washed with DMF for 5 min twice, then with acetone for 2 min twice and finally dried in a jet of air.

After the final coupling cycle, the side-chain protecting groups are cleaved from the amino acids by washing the slide three times for 30 min in a mixture of 51% v/v trifluoroacetic acid, 3% v/v triisobutylsilane (ultrapure, Sigma, USA), 44% v/v DCM and 2% v/v H_2_O. Next, the slide was washed twice with DCM for 5 min and then with 5% DIPEA in DMF for 5 min. Finally, the slide was washed twice for 5 min each in DMF, subsequently in methanol twice for 5 minand then the substrate was dried in an air flow.

### Typical duration of synthesis for one layer

The structuring process for 10,000 spots with 20 different donor slides on one acceptor slide currently requires <15 min: the transfer of one spot currently requires <10 ms (currently 3–10 ms, actual lasing time is 1–5 ms), which sums up to <100 s for 10,000 spots. The exchange of one donor slide for another currently requires about 40 s, which sums up to 800 s for 20 donor slides. Compared with this, the actual lasing time is very short. Therefore, we can easily increase the number of spots to 100,000 with only modest increase in process duration (<30 min). This is certainly not the end of optimization: it should be possible to decrease the donor slide handling time and the laser transfer time as well, by at least 50%. For the repetitive coupling yield and mass spectrometry experiment, lasing duration was 23 min for ∼215,000 spots with 100 μm pitch and 5 ms lasing time per spot.

We only need one coupling step for all 20 amino acids in one layer, owing to the separation of transfer and chemical coupling reaction. Thus, the time required for one layer includes (1) the sequential physical patterning of the 20 different types of amino acid spots (<30 min for 100,000 spots) and (2) one coupling step (60 min at 90 °C). Next, the slide is shortly washed in acetone (<15 min) and the patterning and coupling step is repeated once before the transient Fmoc deprotection to increase the coupling efficiency (that is, double coupling). Thus, elongating all peptides on the acceptor surface by one layer with a pattern of all 20 amino acids, including coupling (twice), wet chemistry capping and Fmoc deprotection requires <5 h.

As structuring, which is rather time efficient, is separate from chemical coupling reaction, which is much more time intensive, it is easily possible to massively increase the throughput by conducting the coupling reaction and chemical washing steps in parallel with many acceptor slides at once. This is probably the most important factor, making our process more time efficient than other approaches. However, this is hard to quantify, as it depends on the actual cLIFT structuring time per array. We can currently process five acceptor slides in parallel in a chemical washing chamber.

### Activation reaction

After the patterning of the different materials, the reaction was initiated by heating the acceptor slide in an oven to 90 °C for 90 min under argon atmosphere. To block (that is, acetylate) the remaining free NH_2_ groups on the acceptor slide, it was incubated in a mixture of acetic anhydrate (10%)+DIPEA (20%)+DMF (70%) for 30 min. Next, the slide was washed with DMF for 5 min twice. Deprotection of the NH_2_ groups of the amino acids was achieved by washing the slide in a solution of piperidine (20%) and DMF (80%) for 20 min. Afterwards, the acceptor slide was washed twice with DMF for 5 min, twice with acetone for 2 min and then dried in a jet of air. The slide was then incubated in PBS with 0.05% Tween 20 (PBS-T) for 15 min. To stain the free amino groups, the slide was incubated with a rhodamine *N*-hydroxysuccinimide ester (5/6-carboxy-tetramethyl-rhodamine succinimidyl ester), diluted 1:10,000 in PBS-T and afterwards washed three times in PBS-T.

### Staining protocol with proteins

For fluorescent staining, we use monoclonal mouse anti-HA antibodies (by Dr G. Moldenhauer, German Cancer Research Center) conjugated with a Cy5 fluorescent dye and monoclonal mouse anti-Flag M2 antibodies (Sigma) conjugated with a Cy3 dye. First, the slide is incubated in PBS-T for 15 min. Next, the surface is blocked in Rockland infrared blocking buffer (MB-070, Rockland Immunochemicals, USA) for 30 min and then washed with PBS-T for 1 min. Staining was performed for 1 h with a mixture of 10% Rockland blocking buffer (500 μl) in PBS-T (4,500 μl), adding 1:1,000 anti-HA and anti-Flag antibodies (5 μl each). Finally, the slide was washed with PBS-T three times for 3 min, briefly rinsed with distilled water and dried in a jet of air.

For the staining of the biotinylated peptides, Alexa Fluor 550-labelled streptavidin was used. The following steps were performed: the slide was washed with PBS-T for 15 min and then blocked with Rockland blocking buffer for 30 min. Afterwards, it was washed with PBS-T for 1 min and stained for 1 h in a mixture of 10% Rockland blocking buffer in PBS-T, and Alexa Fluor 550-labelled streptavidin, diluted 1:5,000, was added. Finally, the slide was washed with PBS-T three times for 3 min and washed with distilled water for 2 min.

### Image acquisition

Fluorescent image acquisition was performed with two different fluorescent scanners: (1) a Molecular Devices (USA) Genepix 4000B fluorescent scanner at the wavelengths 532 and 635 nm with a laser power of 100%, a resolution of 5 μm and a photo multiplier gain of 470; (2) an Innopsys (France) InnoScan 1100 AL at the wavelengths 532 and 635 nm with a low laser power, the resolution set to 5 μm and a photo multiplier gain of 2. Surface topography was measured with a phase-shift interferometer Contour GT (Bruker, USA).

### Data availability

The data that support the findings of this study are available as Supplementary Information and from the corresponding authors on request.

## Additional information

**How to cite this article**: Loeffler, F. F. *et al.* High-flexibility combinatorial peptide synthesis with laser-based transfer of monomers in solid matrix material. *Nat. Commun.* 7:11844 doi: 10.1038/ncomms11844 (2016).

## Supplementary Material

Supplementary Information Supplementary Figures 1-15, Supplementary Tables 1-4, Supplementary Methods and Supplementary References

Supplementary Movie 1 The movie shows the automated cLIFT-system lasing setup. A Prior Scientific PL-200 slide loader automatically handles the substrates and deposits them onto a microscope stage. The acceptor slide is fixed by vacuum. A microscope camera determines the slide position by locating two reference markers on the slide. Donor slides are placed on top of the acceptor slide. The material transfer is actuated by a 1 W, 532 nm cw DPSS laser, switched by an acousto-optic modulator, the laser beam is deflected by a scan head, according to the programmed pattern. Different materials are applied by consecutively transferring from different donor slides. The slide loading system allows for a fully automated patterning process.
