# Supplementary material for: High-flexibility combinatorial peptide synthesis with laser-based transfer of monomers in solid matrix material
Source: Nat Commun. 2016 Jun 14;7:11844. doi: 10.1038/ncomms11844 (PMC4911634; doi:10.1038/ncomms11844)
Supplement: Supplementary Information — Supplementary Figures 1-15, Supplementary Tables 1-4, Supplementary Methods and Supplementary References [file ncomms11844-s1.pdf]

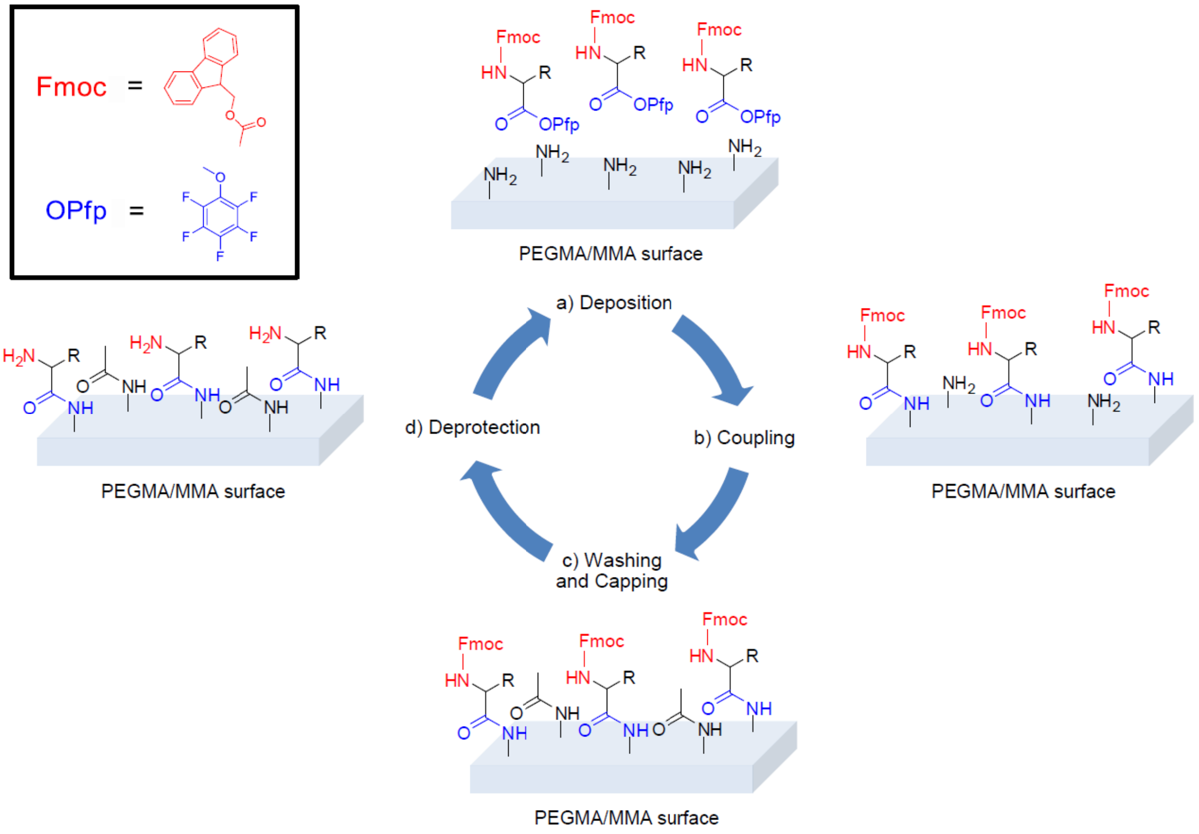

Supplementary Figure 1 *Cycle of the solid phase peptide synthesis. (a) Deposition: activated amino acid derivatives embedded in matrix material are transferred by laser radiation. (b) Coupling: induced by a heating step, the activated amino acid derivatives diffuse and couple to the free amine groups on the surface under inert atmosphere at 90 °C. (c) Washing and capping: uncoupled amino acid derivatives and matrix material are removed in a washing step, followed by capping unreacted free amines with acetic anhydride. (d) Deprotection: the Fmoc protecting groups are cleaved to render free amines on the surface for the next synthesis cycle.*

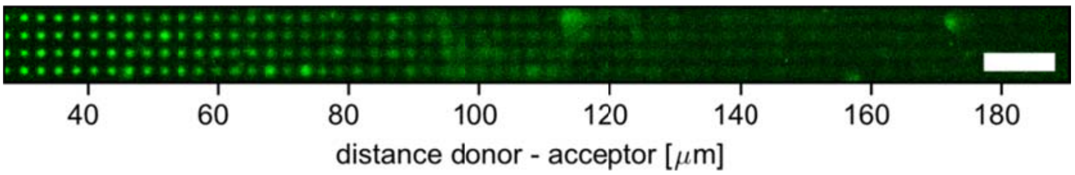

Supplementary Figure 2 *Effect of an increasing gap between donor and acceptor slide on material transfer. The donor slide has been placed onto the acceptor slide such that their edges touch at the left end and are separated by a 250 µm thick spacer on their right end. A*

uniform pattern of spots with an activated biotin building block was transferred to the slide. In the fluorescent image, weakly stained spots are visible up to a distance of about 100 µm.

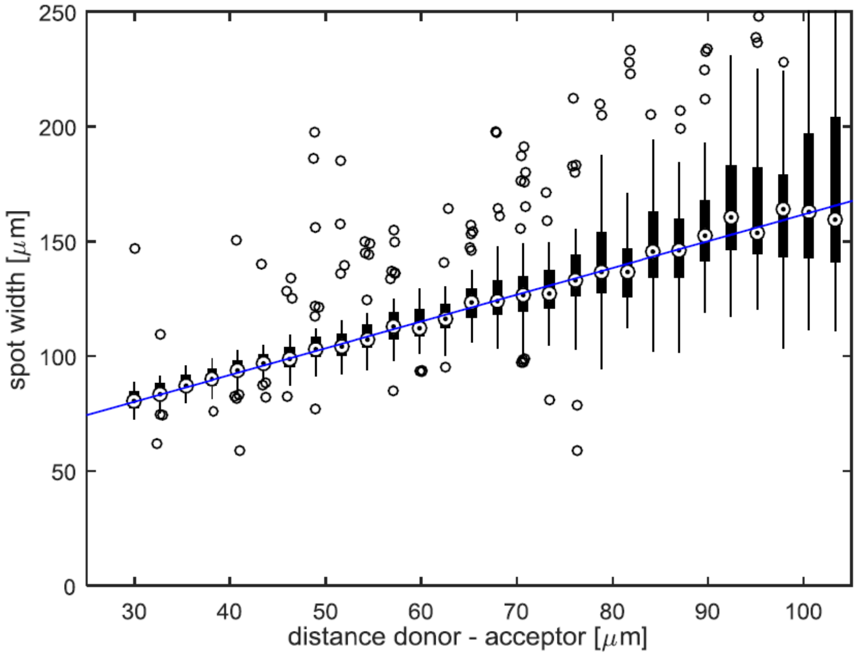

Supplementary Figure 3 *Statistical analysis of the spot width in Supplementary Figure 2 as a function of the gap between the donor and acceptor slide. The larger the gap becomes, the larger is the variation in the transferred spot width.*

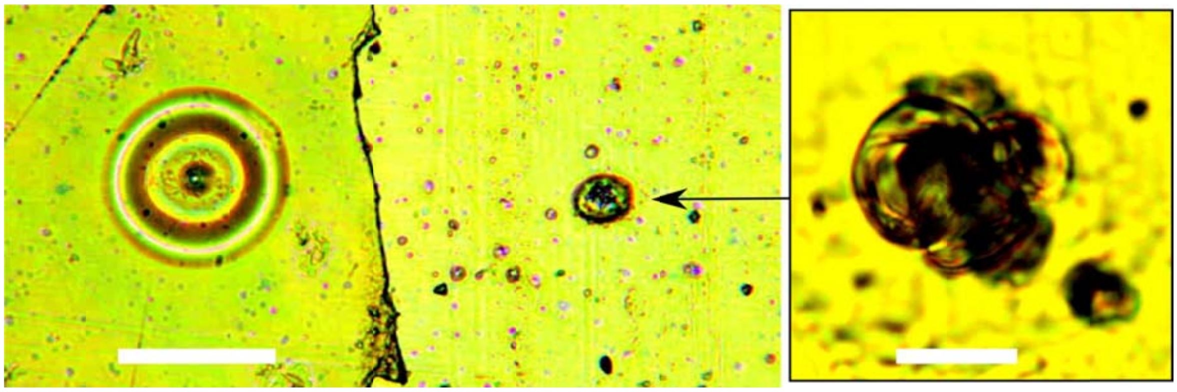

Supplementary Figure 4 *Effect of very intensive laser irradiation of the donor slide. The coated transfer material layer forms a molten ring around the irradiation spot, as can been seen on the left*

side of the microscope image (reflected light image, scale bar 100 µm). For the right half of the picture, showing a second irradiation spot, the material transfer layer was removed mechanically so that the polyimide absorption layer can be analyzed. The laser burns the polyimide and generates blisters (see magnified detail, transmitted light image, scale bar 10 µm). This phenomenon does not occur at lower lasing energies and, thus, determines the upper limit of the feasible parameter combinations. The corresponding transferred material spot on the acceptor slide is of an approximate diameter of 200 µm (not shown).

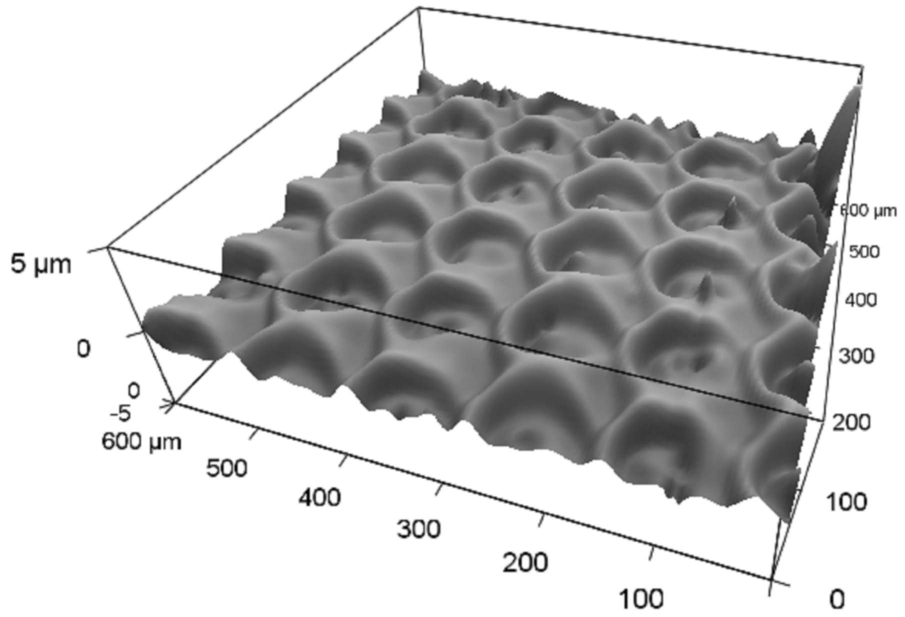

***Supplementary Figure 5** Donor slide surface after the cLIFT process using intensive laser radiation as in Supplementary Fig. 4, analyzed with atomic force microscopy. A crater pattern was generated on the donor slide with constant laser parameters.*

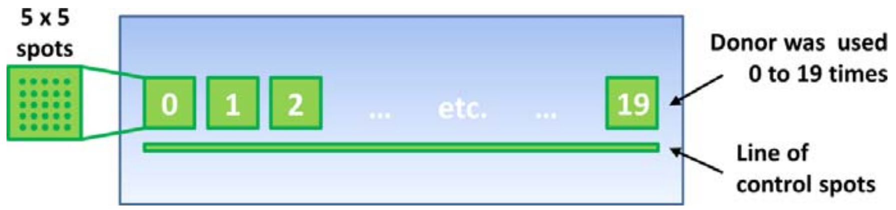

***Supplementary Figure 6** Layout of the donor slide preparation. The donor was irradiated up to 19 times. A line of spots serves as a control.*

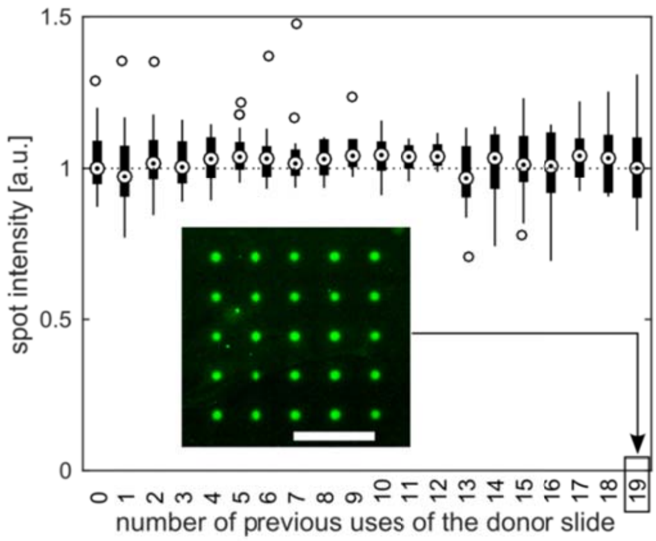

Supplementary Figure 7 *Results of multiple usage of one donor slide, covered with a polymer-glycine layer. The donor slide was positioned with reference markers, to exactly reuse the same positions of the donor slide up to 20 times (scale bar 500 µm).*

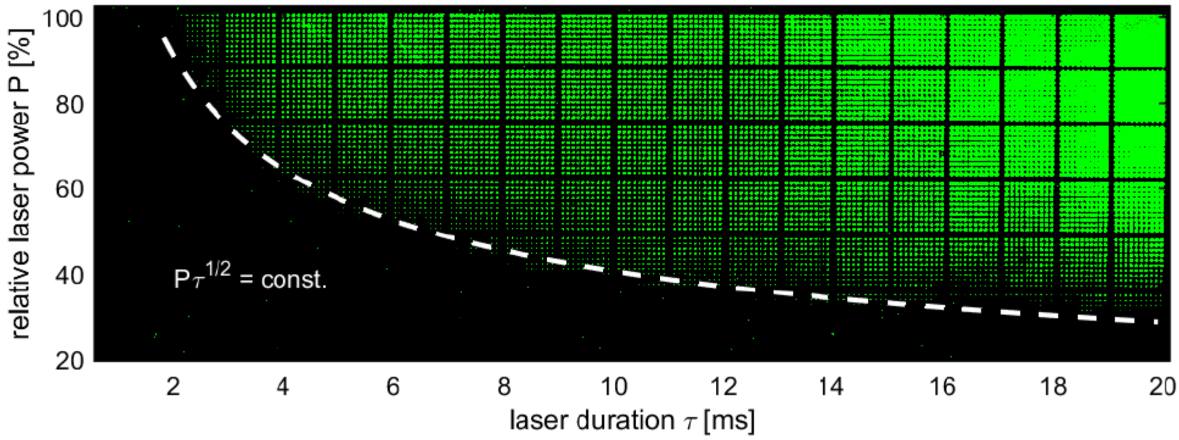

Supplementary Figure 8 *Fluorescence image of a parameter variation pattern. Leucine monomers were transferred, coupled, and stained with a rhodamine* (NHS)-ester. *The laser parameter settings for the transfer of the respective spots are approximated on the axes. The contrast was enhanced such that all spots are visible and the boundary of the feasible region of the parameter space can be derived. The dashed line describes this boundary, derived from physical considerations.*

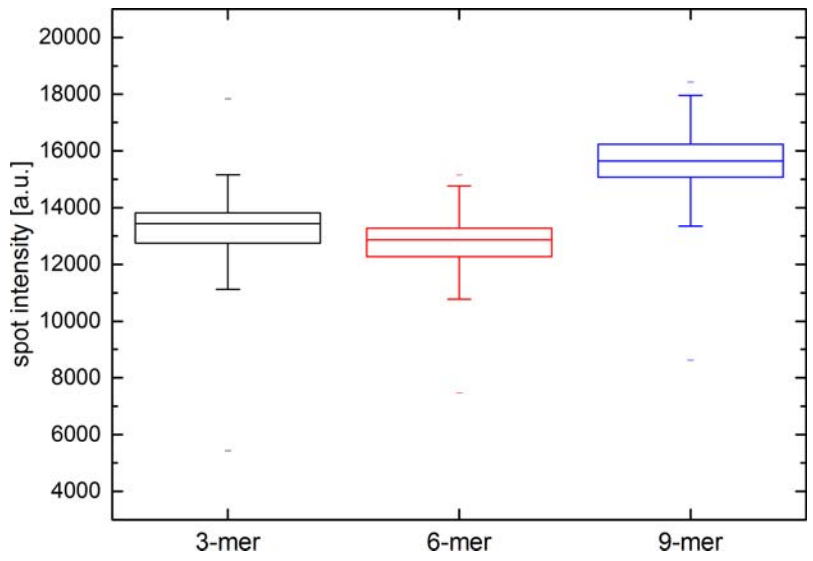

Supplementary Figure 9 *Statistical analysis of spot intensity of the experiment shown in Fig. 4a (3-, 6-, 9-mer peptides). For the experiment shown in Fig. 4a, we analyzed the fluorescence intensity of 1600 spots of each synthesized 3-, 6-, and 9-mer peptide array. No significant difference in fluorescence spot intensity can be observed. Standard deviation is about 10 %. Outliers are mainly caused by surface artefacts.*

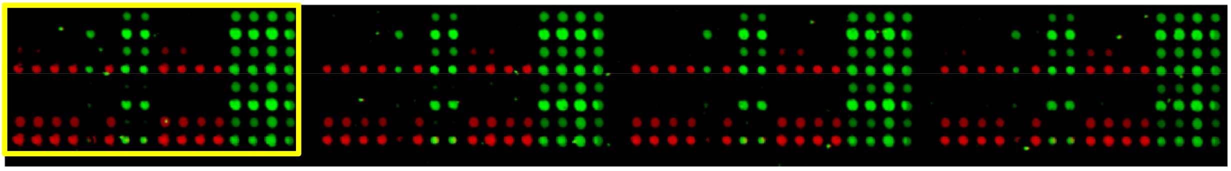

Supplementary Figure 10 *Synthesis of Flag and HA peptides and 62 different variants with the cLIFT system. The left of the four array replicates is highlighted in yellow.*

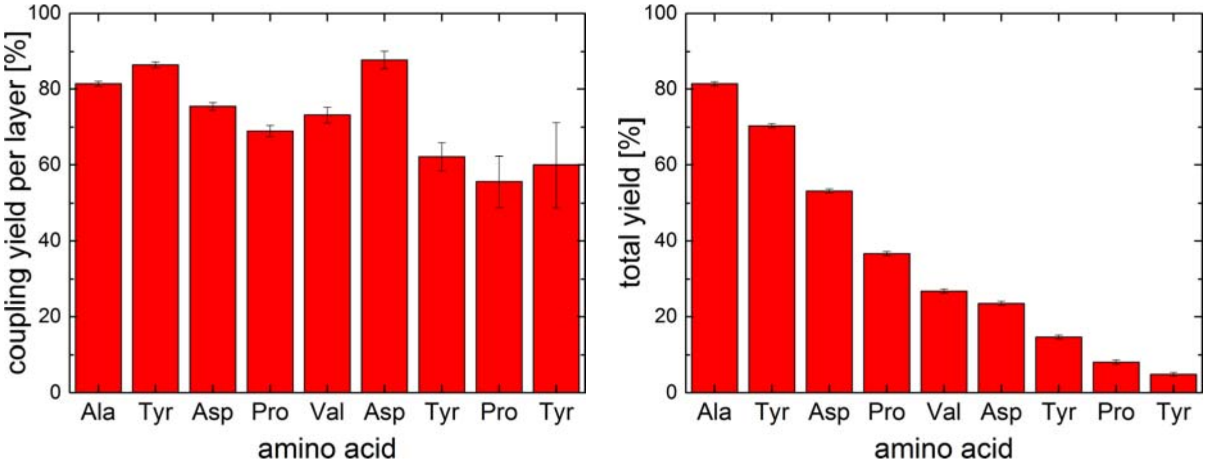

Supplementary Figure 11 *Repetitive coupling yield per layer (left) and total (right). Each coupling yield was determined by calculating the average of four equally treated slides (error bars s.d.).*

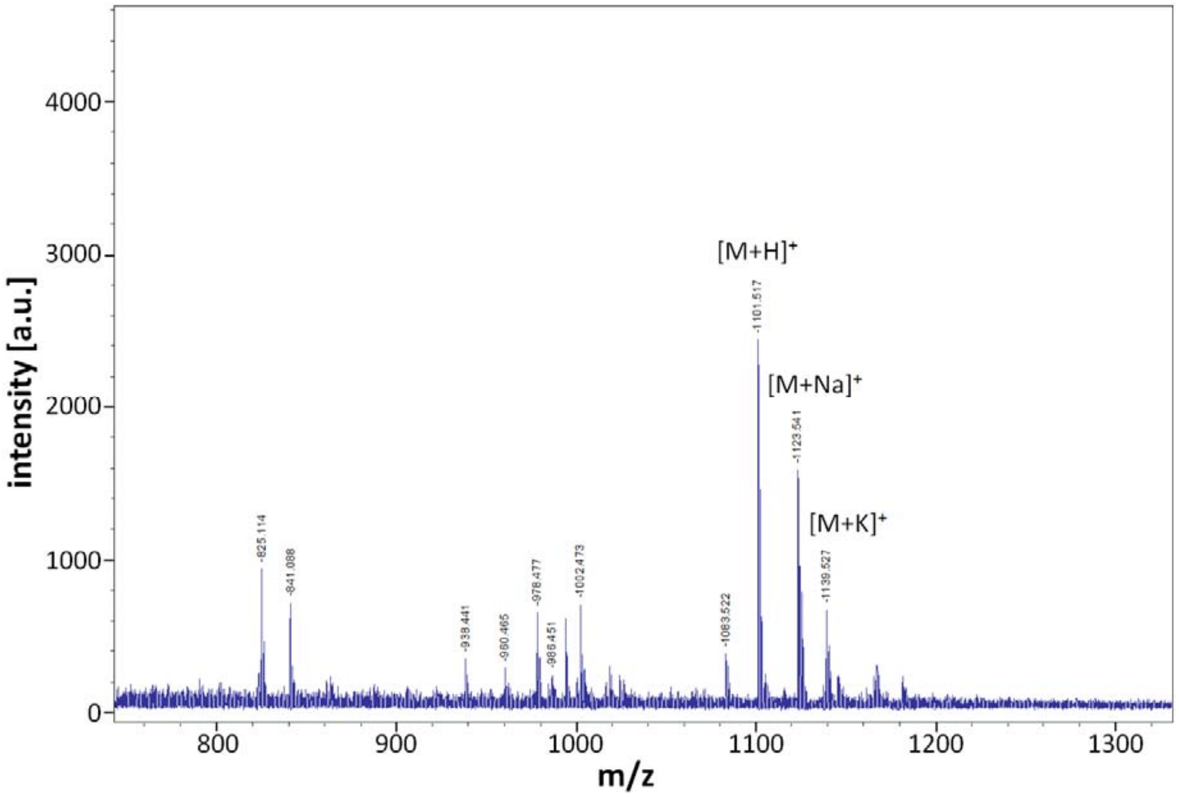

Supplementary Figure 12 *Mass spectrometry analysis of the synthesized HA peptide.*

YPYDVPDYA-amide: m/z (monoisotopic) = 1101.52 (calc. 1101.49)

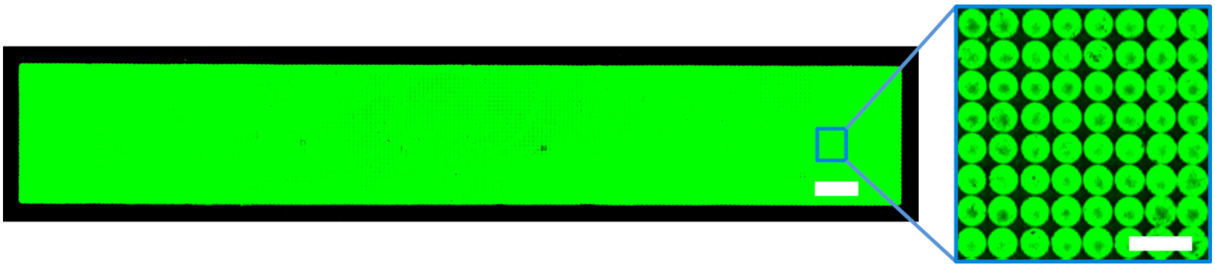

Supplementary Figure 13 *Control synthesis and staining of HA peptide (no Rink amide linker). 200 x 31 spots were synthesized with a pitch of 100 µm on 20.0 mm x 3.1 mm (scale bar 1 mm). In the detail (right, scale bar 200 µm), we increased the contrast and could observe that the spots do not overlap. Moreover, the total area which is covered by spots is only between 56 – 78 %, which causes a lower total coupling yield.*

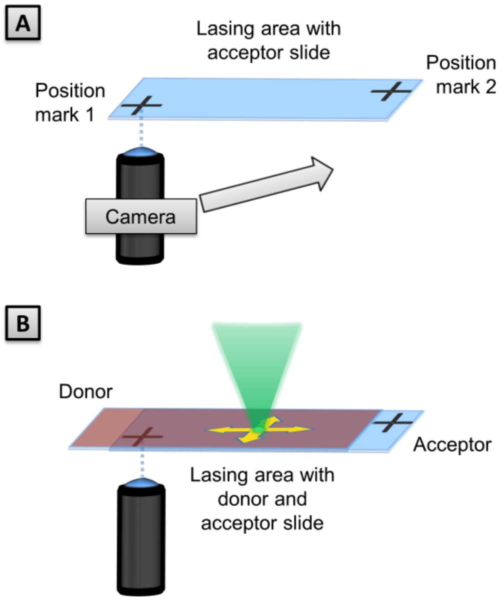

Supplementary Figure 14 *Alignment of slides in the lasing area of the cLIFT system. (A) First, the donor slide is automatically positioned in the lasing area. The exact lateral position of the acceptor slide is recorded by a camera, which measures the position of two position marks on the acceptor slide. The laser focus coordinate system is adjusted accordingly. (B) Fine lateral positioning of the donor slide on top of the acceptor slide is not necessary. An offset of the donor to the acceptor slide is required for the gripper of the automatic slide loading system to handle each slide individually.*

Supplementary Figure 15 *Reference markers on the acceptor surface. 25 circles with a pitch of 100 µm are located on the surface, serving as one reference mark (scale bar 100 µm). The actual position is calculated by determining the center of every circle and determining the weighted average of all circle centers.*

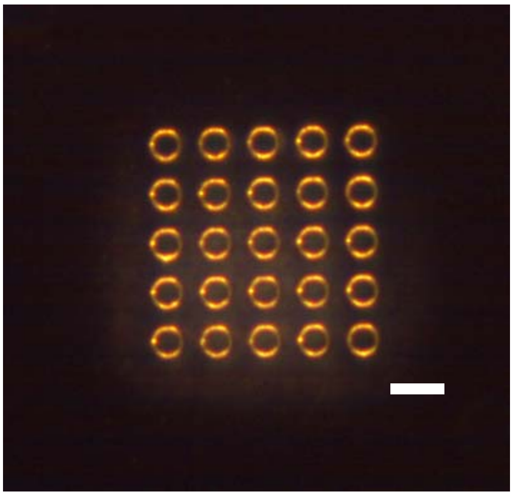

Supplementary Table 1 *Influence of amino acid substitutions on the binding of the monoclonal mouse anti-HA antibody to an altered HA-epitope.*

**amino acid #**		1	2	3	4	5	6	7	8	9	
**HA-epitope**	N-	Tyr	Pro	Tyr	Asp	Val	Pro	Asp	Tyr	Ala	-C
**Good binding**			Asp		Lys						
**Weaker or no binding**						Asp	Asp		Asp	Lys	

Supplementary Table 2 *Influence of amino acid substitutions on the binding of the monoclonal mouse anti-Flag antibody to an altered Flag-epitope*

**amino acid #**		1	2	3	4	5	6	7	8	9	
**Flag-epitope**	N-	Tyr	Asp	Tyr	Lys	Asp	Asp	Asp	Asp	Lys	-C
**Good binding**			Pro			Val				Ala	
**Weaker or no binding**					Asp		Pro		Tyr		

Supplementary Table 3 *Average and standard deviation of the HA and Flag peptide fluorescence signals.*

	**532 nm channel**		**635 nm channel**	
**Peptide**	**Average**	**SD**	**Average**	**SD**
**Background**	660.0	191.9	35.7	10.7
**YDYKDDDDK**	15567.3	3808.1	37.2	3.1
**YPYDVPDYA**	634.5	305.8	5232.5	1298.0

Supplementary Table 4 *Average and standard deviation of the 64 HA and Flag peptide variants' raw fluorescence intensity signals.*

Peptide	532 nm channel Average	532 nm channel SD	635 nm channel Average	635 nm channel SD
YDYDVPDYA	621.8	135.8	5901.2	751.8
YDYDDPDYA	619.8	111.0	5406.6	643.3
YPYKVPDYA	629.9	31.3	4355.9	515.1
YPYDVPDYA	632.3	41.3	4326.4	462.3
YDYDDDDYA	605.5	15.1	4106.0	505.0
YDYDVDDYA	600.3	16.5	3925.0	437.2
YPYKVDDYA	890.2	891.5	3262.9	335.5
YPYDVDDYA	635.9	24.0	2866.5	448.2
YDYDVPDYK	815.4	596.2	1476.3	88.4
YPYDVPDYK	640.8	28.8	1222.5	103.7
YDYDDPDYK	614.6	13.8	902.9	104.2
YPYKVPDYK	638.2	22.8	744.6	142.0
YDYDVDDYK	606.4	17.9	382.9	38.0
YPYKDPDYA	7997.0	1981.7	328.2	56.8
YPYDDPDYA	706.0	63.7	309.4	66.5
YPYDVDDYK	633.0	15.7	257.6	38.2
YDYKVPDYA	9956.1	1941.2	158.2	44.6
YPYKDDDYA	14329.4	1721.1	106.2	14.9
YDYDDDDYK	617.5	13.1	96.4	19.3
YPYDDDDYA	5188.5	1204.9	82.2	17.2
YPYKVDDYK	637.8	9.2	75.3	14.2
YDYKDPDYA	11667.5	6343.6	73.2	30.0
YDYDDDDDK	836.6	589.0	48.1	35.4
YDYDDDDDA	911.8	694.0	46.8	28.4
YPYDDPDYK	627.5	24.5	44.9	4.7
YDYDDPDDA	647.9	19.6	44.0	3.5
YDYKVDDYA	14607.1	1658.0	43.9	5.3
YDYKDDDDA	22678.9	7361.8	42.0	11.3
YPYKVDDDA	881.9	417.0	40.7	11.0
YDYKVPDYK	3196.6	570.8	40.0	3.3
YPYKVPDDA	787.1	106.8	39.3	4.5
YDYKDDDYA	14796.3	4669.4	39.1	5.1
YPYKDPDDA	12948.9	2115.1	39.1	2.1
YPYKDDDDA	22647.4	2716.2	39.0	1.6
YDYKVDDDA	26003.3	3340.3	38.5	1.5
YPYDVPDDA	765.9	38.3	38.2	1.3
YDYKVPDDA	22834.8	4896.5	37.8	1.4
YPYDVDDDA	728.3	34.4	37.6	4.0
YPYDDDDDA	6182.3	1009.7	36.9	1.5
YDYKDPDDA	22724.5	11206.5	36.8	2.2
YPYKDPDYK	759.8	74.4	36.7	1.8
YDYKDPDYK	6657.5	4242.2	36.6	2.4
YPYDDPDDA	933.4	99.3	36.5	1.1
YDYKVDDYK	8078.7	623.1	36.4	1.1
YDYDVPDDA	649.3	30.6	36.3	2.1
YDYKDDDYK	9253.8	2059.7	36.2	1.1
YPYDDDDYK	646.7	28.1	36.0	1.3
YPYKDDDYK	4641.2	531.9	35.9	0.8
YDYKVDDDK	8392.8	779.0	35.9	1.6
YDYKDDDDK	9772.1	2461.1	35.9	1.4
YPYDVPDDK	651.1	26.1	35.7	1.2
0 (Background)	671.5	20.9	35.7	1.1
YDYKDPDDK	8296.8	4865.1	35.7	1.5
YDYDVPDDK	620.6	41.3	35.6	1.4
YPYKDDDDK	3799.7	363.0	35.5	1.2
YPYKVPDDK	636.0	25.4	35.5	0.9
YDYKVPDDK	4634.4	1118.4	35.4	0.8
YPYDDPDDK	629.0	21.7	35.3	1.0
YDYDVDDDA	657.1	16.5	35.2	0.8
YPYKVDDDK	635.8	20.0	35.2	0.9
YPYKDPDDK	738.9	71.0	35.2	0.4
YPYDVDDDK	626.0	14.0	34.8	1.2
YDYDDPDDK	618.4	20.4	34.8	0.5
YDYDVDDDK	606.5	19.2	34.7	0.9
YPYDDDDDK	628.7	16.5	34.7	1.1

Supplementary Methods

Transfer process over longer distances

We statistically analyzed a larger area of the transfer (shown in Supplementary Figure 2) with increasing distance up to 100 µm (about 75 spots for each distance). The analysis in Supplementary Figure 3 shows an increasing spot width median and a drastically increasing variation with increasing distance. However, transfer is generally conducted without a distance between donor and acceptor slide, which significantly decreases the amount of outliers.

Multiple usage of donor slides

We have analyzed the multiple use of one donor slide by irradiating the exact same donor slide positions up to 20 times. Therefore, we generated different patterns corresponding to the number of repetitive irradiations (Supplementary Fig. 6).

We prepared one donor slide, covered with an Fmoc-Glycine-OPfp polymer material layer, by conducting the laser transfer according to the layout in Supplementary Fig. 6. Afterwards, an acceptor slide was patterned once with this donor slide, according to the complete green pattern in Supplementary Fig. 6. The transferred amino acid pattern was coupled and the surface washed, capped, and stained with a rhodamine (NHS)-ester. No significant decrease in fluorescent signal intensity compared to unused areas of the donor slide could be observed (Supplementary Fig. 7). Thus, we can reuse the donor slides at least up to 20 times. The average standard deviation of spot fluorescence intensity is 11.3 %. During peptide synthesis, we repeat the transfer step once, which renders even lower standard deviations.

Optimum laser transfer conditions

To obtain the optimum process parameters (absorbed power P and duration τ of laser irradiation), we varied these parameters systematically in transfer experiments and thoroughly analyzed the results. Supplementary Figure 8 shows the fluorescence image of a parameter variation pattern. For higher irradiation energies, we obtain larger spots. Our

transfer process is limited by two factors: Too strong lasing destroys the laser absorption layer by burning the polyimide and creates a ring of molten transfer material (see Supplementary Fig. 4). With too weak lasing energies, no transfer occurs. We used the formula:

$$P\tau^{0.5} = 4\pi\sqrt{kTD \cdot \rho cT\pi\sigma^2 D} \qquad (1)$$

to model this boundary condition (dashed line in Supplementary Fig. 8), and found a good match to experimentally obtained data (Supplementary Fig. 8).

Hence, we assume that a successful transfer is possible when the transfer material layer of the donor is heated by laser irradiation above a certain threshold temperature T_{th}, which should be close to the glass transition temperature of about 70 °C of the matrix material[25]. Heat conduction within the donor layers is the governing phenomenon, since the transferred spots are larger than the laser beam diameter. Thus, we can use the inhomogeneous heat equation $\partial_t T(x,t) - a\partial_{xx}^2 T(x,t) = f(x,t)$ to estimate the success of the transfer. $a = k/\rho c_p$ denotes the thermal diffusivity with the heat conductivity k, and $f = q/\rho c_p$ describes the heat source normalized by the heat capacity. The heat is generated by the laser in the time interval $0 \le t \le \tau$ and is locally distributed as $q = \frac{P}{2\pi\sigma^2 D}\exp\left(-\frac{r^2}{2\sigma^2}\right)$, where σ is the beam waist and D the layer thickness. For the approximation of the laser pulse as a Dirac pulse at $x, t = 0$, and for disappearing flux at infinity, the heat equation has, with $\tilde{f} := \frac{\frac{P}{2\pi\sigma^2 D}}{\rho c_p}\sigma\tau$, the analytic solution $T = \tilde{f} \cdot \frac{1}{\sqrt{4\pi a t}}\exp\left(-\frac{r^2}{4at}\right)$, known as the (convolved) heat kernel. We bring it into the inverse form $r(t,T)$ and set $T = T_{th}$. In addition, we set $t \le \tau$, because afterwards the system is only cooling down and the threshold temperature cannot be reached anymore. We obtain a solution, if $\tilde{f} > T_{th}\sqrt{4\pi a\tau}$, thus $P\sqrt{\tau} \ge 4\pi\sqrt{kT_{th}D \cdot \rho c_p T_{th}\pi\sigma^2 D}$. Supplementary Figure 8 shows that this functional relationship is indeed experimentally observed, by comparing a fluorescence image of a parameter variation pattern to such a curve.

Spot fluorescence statistics

The fluorescent staining of the 3-, 6-, and 9mer peptide arrays (Fig. 4a) with a fluorescently labeled streptavidin resulted in a rather low intensity standard deviation (about 10 %). We analyzed for each peptide type 1600 spots (Supplementary Fig. 9).

The synthesis of the Flag and HA peptides (see Fig. 4b-d) and 62 variants resulted in the expected results. Supplementary Fig. 10 shows the detail of the synthesis, where the Flag and HA peptides and 62 variants were synthesized in 4 replicas of 8 x 16 spots (each sequence synthesized as 8 spot replicates). The median standard deviation of a spot type was 9.6 % for the anti-Flag antibody staining and 6.6 % for the anti-HA staining (also see Supplementary Table 4).

The monoclonal anti-Flag antibody mainly recognizes the Asp-Tyr-Lys (DYK, amino acid positions 2-4) sequence[1], the monoclonal anti-HA antibody mainly recognizes the sequence Asp-Tyr-Ala (DYA, amino acid positions 7-9)[4]. The following tables conclude the binding motives and the more important amino acids for binding. However, because in some variants more than one amino acid is altered, the binding is more complex than described in the following tables (Supplementary Tables 1 & 2), which only denote a general tendency (list of all 64 average raw signals and standard deviation in Supplementary Table 4).

Considering the whole array, shown in Fig. 4b, we calculated an average deviation of 24.4 % for the HA peptide staining signal and 24.8 % for the Flag peptide staining (Supplementary Table 3).

Repetitive coupling yield and mass spectrometry

To determine the repetitive coupling yield, we synthesized the HA peptide (sequence: YPYDVPDYA). Therefore, we processed 5 surfaces with 10/90 PEGMA/PMMA coating for the synthesis of the HA peptide. Four surfaces were derivatized with a Rink amide linker (Iris Biotech GmbH, Germany), which allows for cleaving the fully synthesized peptides from the surface after the synthesis. The fifth slide was used for a control synthesis, without the Rink amide linker. For a planar and very dense synthesis, we chose a pattern of >215,000 spots with 100 µm pitch and maximum laser intensity, assuming to cover the whole slide with an

overlapping spot pattern. Thus, we did not use reference markers to position the spot patterns of the consecutive amino acid layers. However, after the HA peptide synthesis, a control staining revealed that the spots did not overlap (Supplementary Fig. 13) and only about 56 – 80 % of the slide was covered uniformly with the amino acid polymer mixture in each layer. This explains why we only achieved an average coupling yield per amino acid of 72.3 ± 11.0 % (Supplementary Fig. 11), in comparison to about 90 % in Stadler *et al*.[1]: the surface was not covered uniformly and without reference markers, the overlap of the patterns of sequential layers was not sufficient. Furthermore, the HA peptide is known to have a difficult amino acid sequence (e.g. contains two prolines), which, according to Stadler *et al*.[1], is known to result in lower average coupling yields of about 83 % from solution.

The fifth surface was used for a control experiment without the Rink amide linker (Supplementary Fig. 13), where the HA peptide was synthesized in a rectangle of 20 mm x 3.1 mm (200 x 31 spots, pitch of 100 µm).

For each layer of amino acid on an acceptor slide, one donor slide was prepared and used (HA peptide: Tyr-Pro-Tyr-Asp-Val-Pro-Asp-Tyr-Ala). Spin-coating was performed as described in the methods section. Patterning and coupling was performed twice (reusing each donor slide once), as described in the methods section, to increase the coupling yield.

Acceptor slides with 10/90 PEGMA/PMMA coating (PEPperPRINT GmbH) were Fmoc deprotected by immersing the slides in a solution of piperidine (20 %) and DMF (80 %) for 20 min. Afterwards, the acceptor slides were washed three times with DMF for 5 min each, then with MeOH for 2 min and 1 min in DCM.

Four slides were derivatized with the Rink amide linker by incubating in a 0.2 M solution of Rink amide linker, Pentafluorophenol, diisopropylcarbodiimde (DIC) in DMF overnight. Afterwards, slides were washed 3 x 5 min in DMF, once for 2 min in MeOH, and 1 min in DCM. To block remaining free NH_2 groups, slides were washed in a mixture of acetic anhydrate (Ac_2O, 10 %), N,N-diisopropylethylamine (DIPEA, 20 %), and DMF (70 %) for 2 h. Then, the slides were washed three times for 5 minutes with DMF, then with MeOH for 2 min and 1 min in DCM.

Each cLIFT step required 23 min per slide (215,172 spots, 5 ms, 100 % laser power), coupling was performed at 94 °C for 60 min, with a subsequent cooling step for 15 min at room

temperature. Then, a short washing step was conducted in the automated wet chemistry machine setup: twice 1 min in acetone with sonification, 1 min in MeOH with sonification. Patterning, coupling, and the short washing step were repeated once for a better yield.

Acetylation ("capping") was performed as described in the methods section. However, we performed the capping step overnight, to ensure that no residual amino groups might interfere with the measurement.

To measure the amount of cleaved Fmoc from the surface with UV spectrometry, surfaces were pre-swelled in DCM for 10 min. Afterwards, 1 mL of Fmoc deprotection solution (20 % piperidine in DMF) was carefully distributed on one acceptor slide. After 20 min, the solution was removed and reused for the next 3 slides. Each surface was washed with 100 µL of this solution, which was also recollected and reused. After deprotection of all four surfaces, this 1 mL of deprotection solution contains the piperidine-fulvene-adducts of four deprotected slides. We used a UV spectrometer at 301 nm to measure absorption and calculated the coupling efficiency, according to Beyer *et al.*[3]. Then, the slides were washed three times for 5 minutes with DMF, then with MeOH for 2 min and 1 min in DCM.

The final volume was measured and the yield was determined via the formula:

$$\text{Yield } [nmol\ cm^{-2}] = \frac{EV \cdot 10^6}{d\varepsilon A} \qquad (2)$$

A = area [cm^2]: 19.76 cm^2 (2.54 cm x 7.62 cm); *V* = volume [mL]; *E* = measured extinction; ε = molar absorption coefficient [L mol^{-1} cm^{-1}]: 5129 L mol^{-1} cm^{-1}; d = distance [cm]: 1 cm

The synthesis procedure was repeated for a total of 9 layers in the following synthesis order (C->N): Ala, Tyr, Asp, Pro, Val, Asp, Tyr, Pro, Tyr. Due to small measured values obtained via UV spectrometry, the coupling efficiencies of the last two amino acids are rather inaccurate.

For the MALDI mass spectrometry experiment, the HA peptides were cleaved from the surface and at the same time side chain protecting groups were removed by swelling the slides 10 min with DCM, followed by 2 hours incubation in a 1 mL solution of 91 % TFA, 4 % DCM, 3 % triisobutylsilane, and 2 % H_2O. The TFA solution, containing the product, was given into a LoBind (Eppendorf) reaction vessel. The TFA solution was evaporated under vacuum, and the product was analyzed by MALDI mass spectrometry (Supplementary Fig. 12).

For the control slide without the Rink amide linker, the side chain protecting groups were removed according to the protocol in the Materials section. The incubation of this control slide with monoclonal mouse anti-HA antibodies conjugated with Cy3 fluorescent dye (Supplementary Fig. 13) was performed as described in the methods section.

A similar experiment was described in the supplementary materials section of Stadler *et al.*[1]. We rely on the same chemistry, including coupling steps of 90°C for 60 min. However, a surface with a much higher amino group loading (100 PEGMA coating) was used in Stadler *et al.*[1]. There, a laser printer is used to print the same type of amino acid derivatives and matrix material in form of solid particles onto the same type of acceptor slides. In the supporting information of this publication, it was shown that the average amino acid coupling efficiency ranges between 90 – 93 % and no racemization could be observed.

The distinct difference to our approach is the laser-based transfer and the amount of material. We have shown in Maerkle *et al.*[2] that we can fuse the aforementioned amino acid particles with strong laser irradiation. There, we showed that irradiation and the resulting heat do not harm the activated amino acid monomers in the matrix material. Thus, our laser-based transfer approach does not seem to change the chemistry in comparison to previously published approaches.

Click reaction

Fmoc-Pra-OPfp was prepared from commercially available Fmoc-Pra-OH via a reaction with pentafluorophenyl tetrafluoroacetate according to Green *et al.*[5].

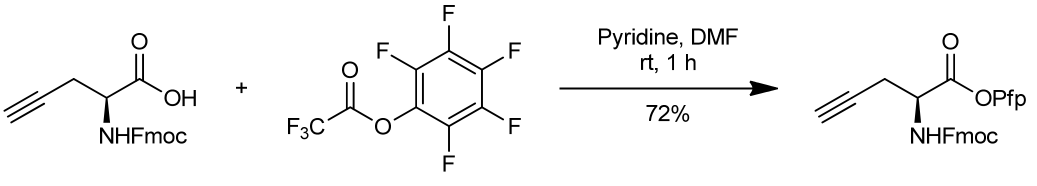

Activation reaction of Fmoc-Pra-OH with pentafluorophenyl tetrafluoroacetate*.*

In a 100 mL round-bottomed flask, 200 mg Fmoc-Propargylglycine (0.596 mmol, 1.00 eq.) was dissolved in 5.00 mL DMF. Under stirring, 205 µL pentafluorophenyl trifluoroacetate (334 mg, 1.19 mmol, 2.00 eq.) and 52.9 µL pyridine (51.9 mg, 0.656 mmol, 1.10 eq.) were added and stirring continued for 1 h at room temperature. The reaction was quenched with

0.1 M HCl (50.0 mL) and the product extracted from the aqueous phase with CH_2Cl_2 (3 x 25.0 mL). The united organic phases were dried over Na_2SO_4 and the solvent removed in vacuo, yielding a white powder (215 mg, 0.429 mg, 72 %).

^{1}H-NMR (300 MHz, $CDCl_3$): δ = 2.22 (t, J = 2.6 Hz, 1 H, CC*H*), 2.87 to 3.12 (m, 2H, C*H₂*), 4.31 (t, J = 7.0 Hz, 1 H, C*H*CO_2R) , 4.51 (d, J = 7.3 Hz, 2 H, Fmoc-CH_2), 4.99 (dt, J = 8.7, 4.8 Hz, 1 H, Fmoc-C*H*), 5.71 (d, J = 9.6 Hz, 1 H, N*H*), 7.36 (t, J = 7.4 Hz, 2 H, Fmoc-H_{ar}), 7.45 (t, J = 7.4 Hz, 2 H, Fmoc-H_{ar}), 7.65 (d, J = 7.6 Hz, 2 H, Fmoc-H_{ar}), 7.82 (d, J = 7.2 Hz, 2 H, Fmoc-H_{ar}) ppm.

^{13}C-NMR (75 MHz, $CDCl_3$): δ = 22.8, 47.1, 52.4, 67.6, 72.9, 120.1, 125.1, 127.1, 127.8, 141.4, 143.7, 155.5 ppm.

^{19}F-NMR (282 MHz, $CDCl_3$): δ = -151.7 (d, J = 17.5 Hz, 2 F), -156.8 (t, J = 21.8 Hz, 1 F), -161.7 (dd, J = 21.5, 17.3 Hz, 2 F) ppm.

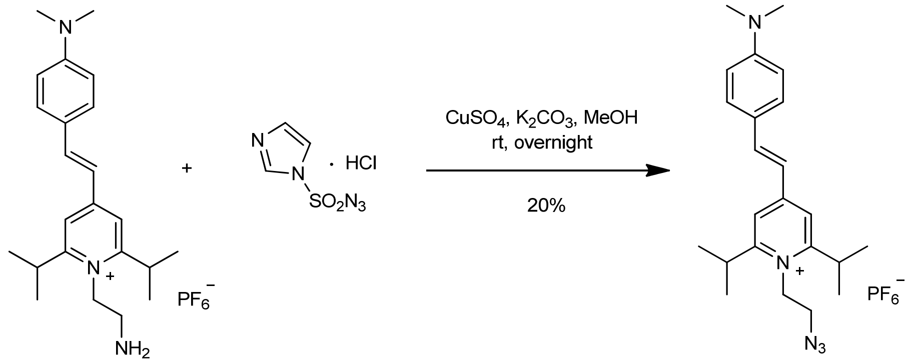

Diazo transfer reaction on styrylpyridinium fluorophores.

To label the patterned Fmoc-Pra-OPfp with a styrylpyridinium dye[6], we used the click reaction. The dye was functionalized with an azide group in a diazo transfer reaction[7] with imidazole-1-sulfonyl azide hydrochloride **5** to yield the corresponding azides (shown above).

In a 25 mL round-bottomed flask, 43.0 mg Styrylpyridinium fluorophore **6-NH₂** (86.0 µmol, 1.00 eq.) was dissolved in 10.0 mL Methanol. 20.0 mg K_2CO_3 (14.5 µmol, 1.67 eq.), $CuSO_4$ x 5 H_2O (6.00 mg, 86.0 µmol, 0.28 eq.) and imidazolesulfonyl azide hydrochloride (29.0 mg, 13.8 µmol, 1.60 eq.) were added and the reaction mixture was stirred overnight at room temperature. Solvents were removed *in vacuo* and the crude product was purified by filtration through a short silica gel plug (3.5 x 3 cm) using acetonitrile as eluent. Evaporation of the solvent yielded a red fluorescent solid (9.00 mg, 17.0 µmol, 20 %).

^{1}H-NMR (300 MHz, $CDCl_3$): δ = 1.46 to 1.54 (m, 12 H, ^{i}Pr-CH_3), 3.13 (s, 6 H, NCH_3), 3.62 (hept, J = 6.8 Hz, 2 H, ^{i}Pr-CH), 4.00 (t, J = 5.7 Hz, 2 H, CH_2), 4.80 (t, J = 5.5 Hz, 2 H, CH_2), 6.76 (d, J = 9.0 Hz, 2 H, H_{ar}), 6.89 (d, J = 16 Hz, 1 H, CH), 7.48 to 7.64 (m, 5H, H_{ar}, CH) ppm.

^{13}C-NMR (75 MHz, $CDCl_3$): δ = 31.5, 40.4, 51.3, 77.4, 112.2, 118.5, 130.8, 142.9, 154.2, 164.1 ppm.

IR (KBr): $\tilde{\upsilon}$ = 2151 (s, N_3) cm^{-1}.

HRMS ($[C_{23}H_{32}N_5]^+$): m/z = 378.2764 (calc. 378.2658).

An experiment with **7-NH$_2$**, in which the amino group is separated from the fluorophore by an additional CH_2 unit, confirmed this assumption with a substantially larger yield. The polarity of the product was considerably lower than of the reactants and could therefore easily be separated by passing it through a short plug of silica using acetonitrile as eluent. This modified fluorophore was now used as a marker to demonstrate successful click reactions on the previously prepared Pra-structured glass slide.

To complete the click reaction, the entire slide was brought into reaction with **6-N$_3$** using $CuSO_4$ / ascorbic acid in DMF for 1 h and after washing and drying, it was scanned.

Supplementary References

1. Stadler, V. *et al*. Combinatorial synthesis of peptide arrays with a laser printer. *Angew. Chem. Int. Edit.* **47** (37), 7132–7135 (2008).
2. Maerkle, F. *et al*. High-density peptide arrays with combinatorial laser fusing. *Adv. Mater.* **26** (22), 3730–3734 (2014).
3. Beyer, M., Felgenhauer, T., Bischoff, F. R. & Breitling, F. A novel glass slide-based peptide array support with high functionality resisting non-specific protein adsorption. *Biomaterials* **27** (18), 3505–3514 (2006).
4. Loeffler, F. *et al*. Biomolecule Arrays Using Functional Combinatorial Particle Patterning on Microchips. *Adv. Funct. Mater.* **22** (12), 2503–2508 (2012).
5. Green, M. & Berman, J. Preparation of Pentafluorophenyl Esters of Fmoc Protected Amino-Acids with Pentafluorophenyl Trifluoroacetate. *Tetrahedron Lett.* **31** (41), 5851–5852 (1990).
6. Rudat, B. *et al*. Novel Pyridinium Dyes That Enable Investigations of Peptoids at the Single-Molecule Level. *J. Phys. Chem. B* **114** (42), 13473–13480 (2010).
7. Goddard-Borger, E. D. & Stick, R. V. An efficient, inexpensive, and shelf-stable diazotransfer reagent: Imidazole-1-sulfonyl azide hydrochloride. *Org. Lett.* **9** (19), 3797–3800 (2007).
